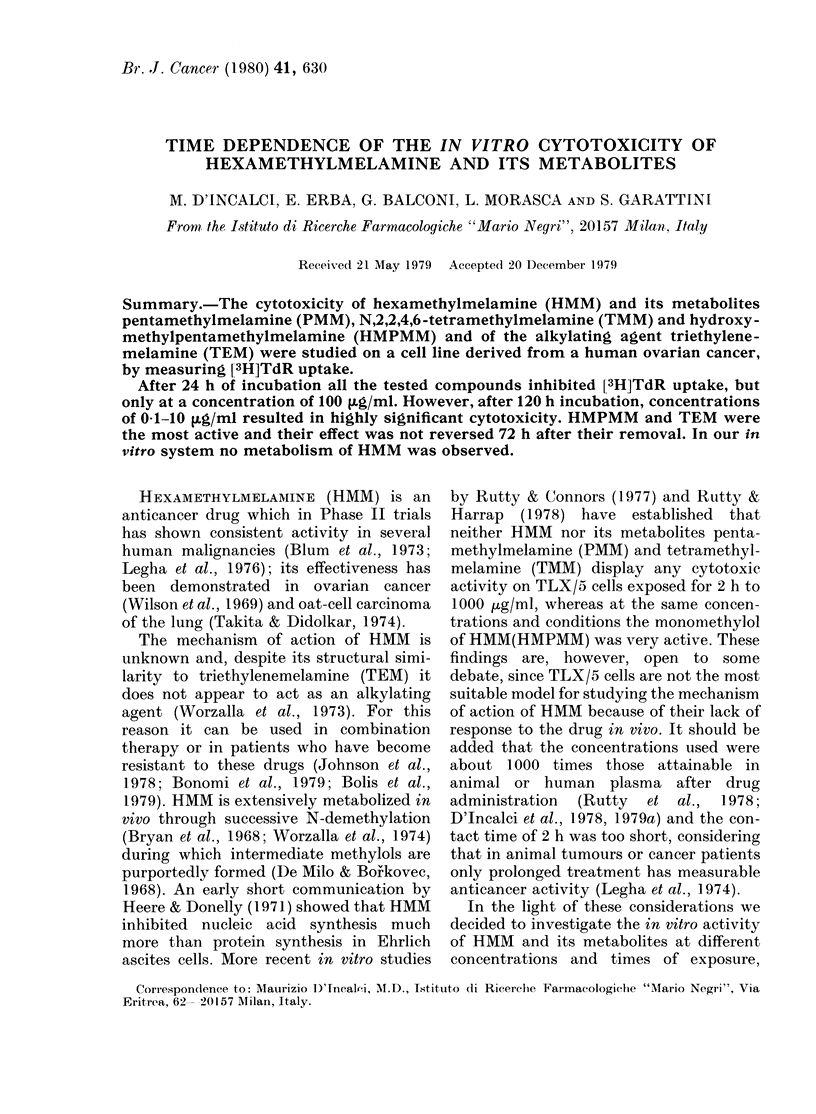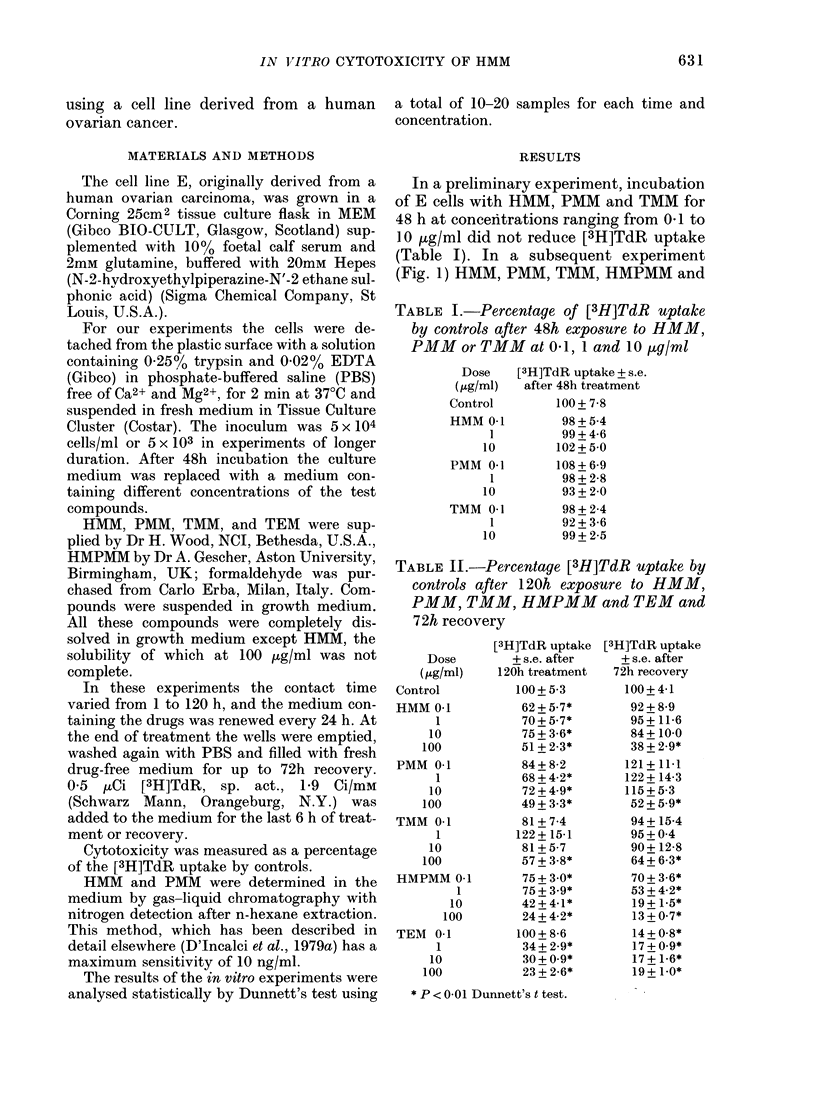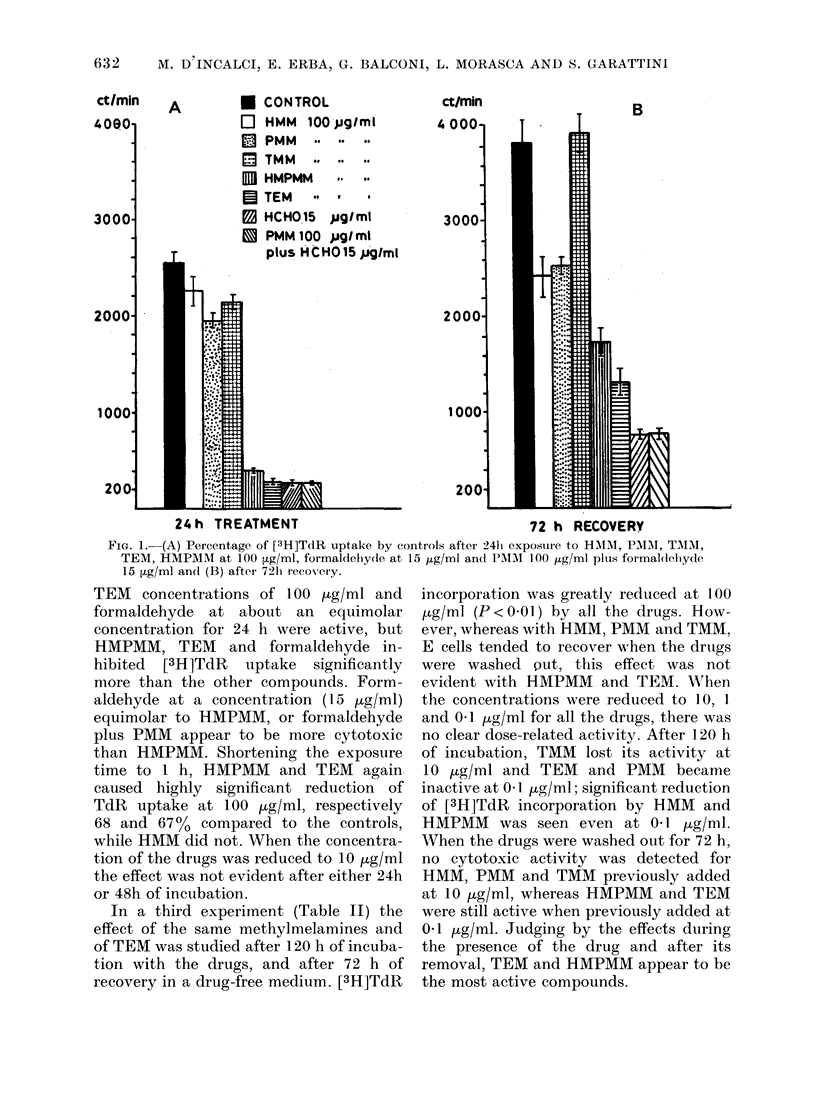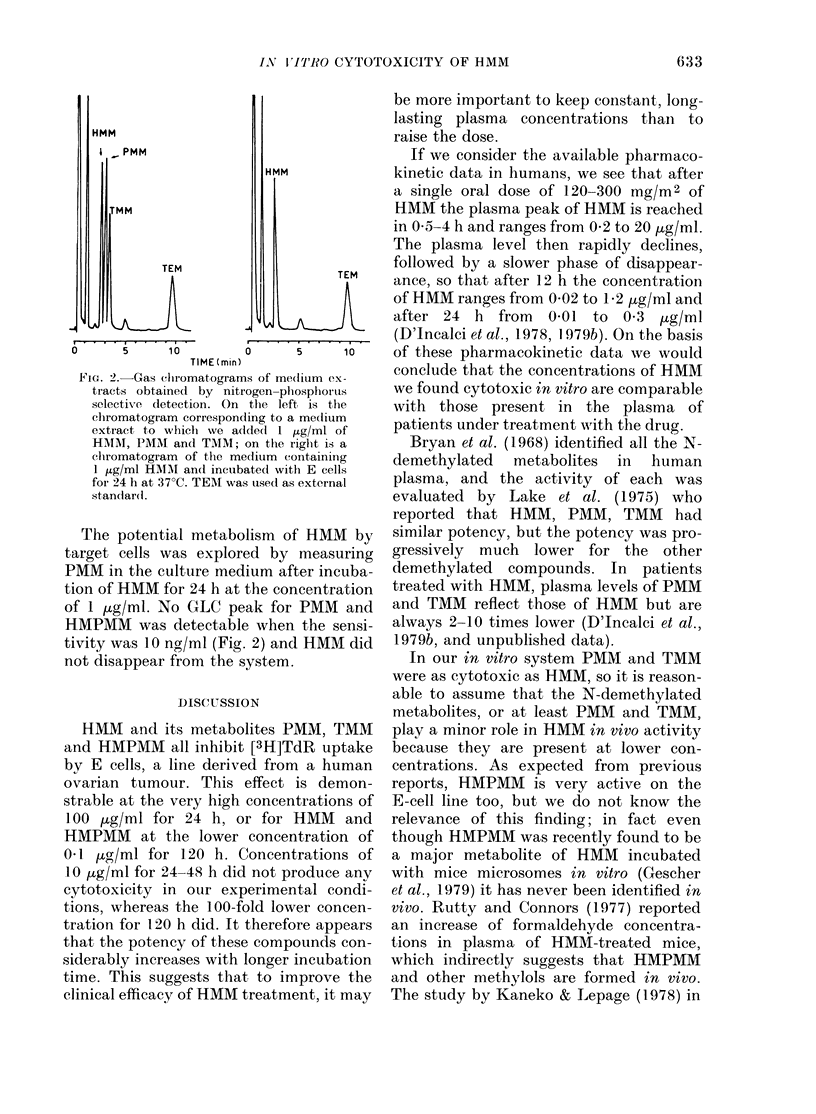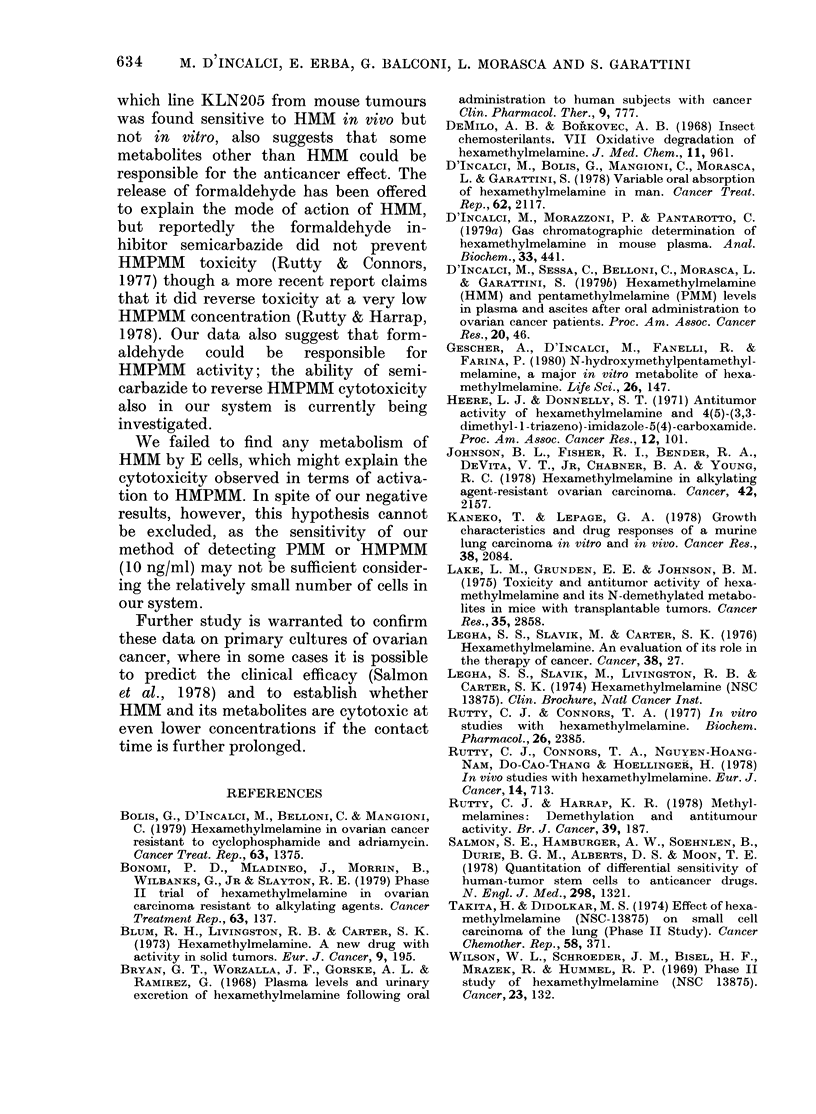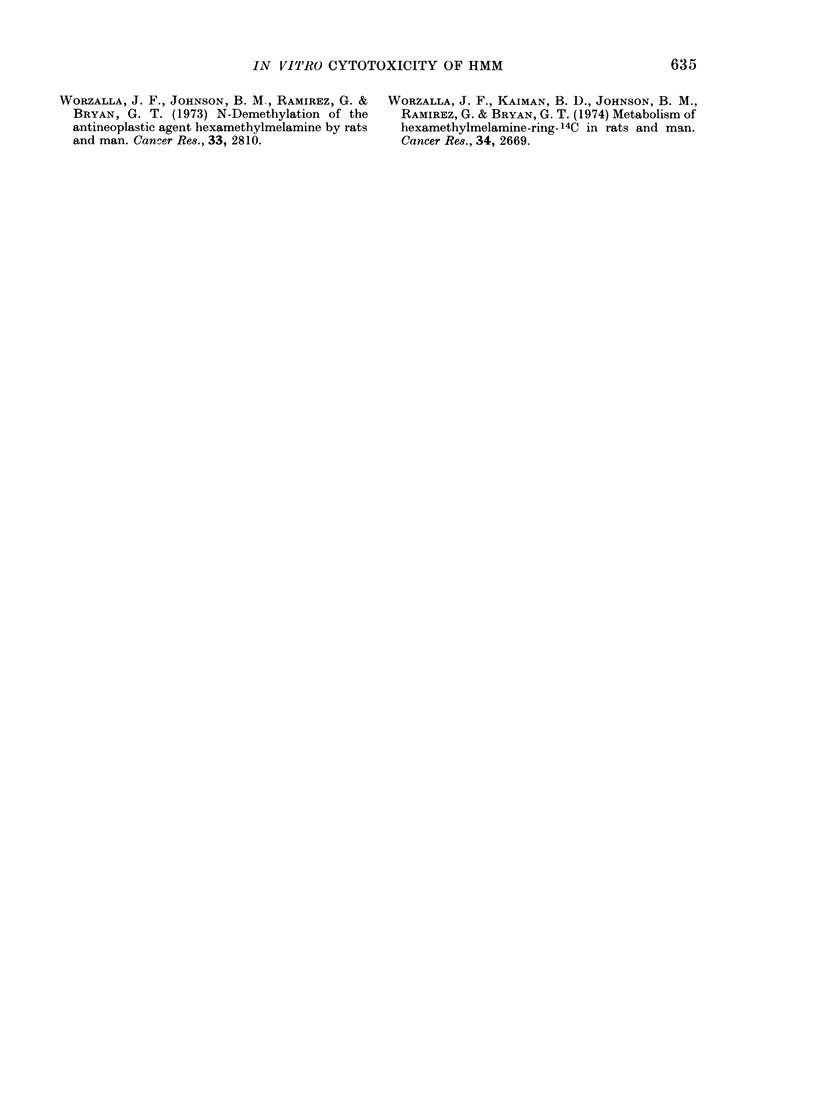# Time dependence of the in vitro cytotoxicity of hexamethylmelamine and its metabolites.

**DOI:** 10.1038/bjc.1980.106

**Published:** 1980-04

**Authors:** M. D'Incalci, E. Erba, G. Balconi, L. Morasca, S. Garattini

## Abstract

The cytotoxicity of hexamethylmelamine (HMM) and its metabolites pentamethylmelamine (PMM), N,2,2,4,6-tetramethylmelamine (TMM) and hydroxymethylpentamethylmelamine (HMPMM) and of the alkylating agent triethylenemelamine (TEM) were studied on a cell line derived from a human ovarian cancer, by measuring [3H]TdR uptake. After 24 h of incubation all the tested compounds inhibited [3H]TdR uptake, but only at a concentration of 100 micrograms/ml. However, after 120 h incubation, concentrations of 0.1--10 micrograms/ml resulted in highly significant cytotoxicity. HMPMM and TEM were the most active and their effect was not reversed 72 h after their removal. In our in vitro system no metabolism of HMM was observed.


*Br. J. Cancer* (1980) **41**, 630

### TIME DEPENDENCE OF THE *IN VITRO* CYTOTOXICITY OF HEXAMETHYLMELAMINE AND ITS METABOLITES

M. D'INCALCI, E. ERBA, G. BALCONI, L. MORASCA AND S. GARATTINI

*From the Istituto di Ricerche Farmacologiche "Mario Negri", 20157 Milan, Italy*

Received 21 May 1979 Accepted 20 December 1979

**Summary.—The cytotoxicity of hexamethylmelamine (HMM) and its metabolites pentamethylmelamine (PMM), N,2,2,4,6-tetramethylmelamine (TMM) and hydroxymethylpentamethylmelamine (HMPMM) and of the alkylating agent triethylenemelamine (TEM) were studied on a cell line derived from a human ovarian cancer, by measuring [$^3$H]TdR uptake.**

**After 24 h of incubation all the tested compounds inhibited [$^3$H]TdR uptake, but only at a concentration of 100 μg/ml. However, after 120 h incubation, concentrations of 0·1–10 μg/ml resulted in highly significant cytotoxicity. HMPMM and TEM were the most active and their effect was not reversed 72 h after their removal. In our *in vitro* system no metabolism of HMM was observed.**

HEXAMETHYLMELAMINE (HMM) is an anticancer drug which in Phase II trials has shown consistent activity in several human malignancies (Blum *et al.*, 1973; Legha *et al.*, 1976); its effectiveness has been demonstrated in ovarian cancer (Wilson *et al.*, 1969) and oat-cell carcinoma of the lung (Takita & Didolkar, 1974).

The mechanism of action of HMM is unknown and, despite its structural similarity to triethylenemelamine (TEM) it does not appear to act as an alkylating agent (Worzalla *et al.*, 1973). For this reason it can be used in combination therapy or in patients who have become resistant to these drugs (Johnson *et al.*, 1978; Bonomi *et al.*, 1979; Bolis *et al.*, 1979). HMM is extensively metabolized *in vivo* through successive N-demethylation (Bryan *et al.*, 1968; Worzalla *et al.*, 1974) during which intermediate methylols are purportedly formed (De Milo & Bořkovec, 1968). An early short communication by Heere & Donelly (1971) showed that HMM inhibited nucleic acid synthesis much more than protein synthesis in Ehrlich ascites cells. More recent *in vitro* studies by Rutty & Connors (1977) and Rutty & Harrap (1978) have established that neither HMM nor its metabolites pentamethylmelamine (PMM) and tetramethylmelamine (TMM) display any cytotoxic activity on TLX/5 cells exposed for 2 h to 1000 μg/ml, whereas at the same concentrations and conditions the monomethylol of HMM(HMPMM) was very active. These findings are, however, open to some debate, since TLX/5 cells are not the most suitable model for studying the mechanism of action of HMM because of their lack of response to the drug *in vivo*. It should be added that the concentrations used were about 1000 times those attainable in animal or human plasma after drug administration (Rutty *et al.*, 1978; D'Incalci *et al.*, 1978, 1979*a*) and the contact time of 2 h was too short, considering that in animal tumours or cancer patients only prolonged treatment has measurable anticancer activity (Legha *et al.*, 1974).

In the light of these considerations we decided to investigate the *in vitro* activity of HMM and its metabolites at different concentrations and times of exposure,

Correspondence to: Maurizio D'Incalci, M.D., Istituto di Ricerche Farmacologiche "Mario Negri", Via Eritrea, 62—20157 Milan, Italy.

using a cell line derived from a human ovarian cancer.

### MATERIALS AND METHODS

The cell line E, originally derived from a human ovarian carcinoma, was grown in a Corning 25cm$^2$ tissue culture flask in MEM (Gibco BIO-CULT, Glasgow, Scotland) supplemented with 10% foetal calf serum and 2mM glutamine, buffered with 20mM Hepes (N-2-hydroxyethylpiperazine-N′-2 ethane sulphonic acid) (Sigma Chemical Company, St Louis, U.S.A.).

For our experiments the cells were detached from the plastic surface with a solution containing 0·25% trypsin and 0·02% EDTA (Gibco) in phosphate-buffered saline (PBS) free of $Ca^{2+}$ and $Mg^{2+}$, for 2 min at 37°C and suspended in fresh medium in Tissue Culture Cluster (Costar). The inoculum was $5 \times 10^4$ cells/ml or $5 \times 10^3$ in experiments of longer duration. After 48h incubation the culture medium was replaced with a medium containing different concentrations of the test compounds.

HMM, PMM, TMM, and TEM were supplied by Dr H. Wood, NCI, Bethesda, U.S.A., HMPMM by Dr A. Gescher, Aston University, Birmingham, UK; formaldehyde was purchased from Carlo Erba, Milan, Italy. Compounds were suspended in growth medium. All these compounds were completely dissolved in growth medium except HMM, the solubility of which at 100 μg/ml was not complete.

In these experiments the contact time varied from 1 to 120 h, and the medium containing the drugs was renewed every 24 h. At the end of treatment the wells were emptied, washed again with PBS and filled with fresh drug-free medium for up to 72h recovery. 0·5 μCi [$^3$H]TdR, sp. act., 1·9 Ci/mM (Schwarz Mann, Orangeburg, N.Y.) was added to the medium for the last 6 h of treatment or recovery.

Cytotoxicity was measured as a percentage of the [$^3$H]TdR uptake by controls.

HMM and PMM were determined in the medium by gas–liquid chromatography with nitrogen detection after n-hexane extraction. This method, which has been described in detail elsewhere (D'Incalci *et al.*, 1979*a*) has a maximum sensitivity of 10 ng/ml.

The results of the *in vitro* experiments were analysed statistically by Dunnett's test using a total of 10–20 samples for each time and concentration.

### RESULTS

In a preliminary experiment, incubation of E cells with HMM, PMM and TMM for 48 h at concentrations ranging from 0·1 to 10 μg/ml did not reduce [$^3$H]TdR uptake (Table I). In a subsequent experiment (Fig. 1) HMM, PMM, TMM, HMPMM and

TABLE I.—*Percentage of [$^3$H]TdR uptake by controls after* 48*h exposure to HMM, PMM or TMM at* 0·1, 1 *and* 10 *μg/ml*

| Dose (μg/ml) | [$^3$H]TdR uptake ± s.e. after 48h treatment |
|---|---|
| Control | 100 ± 7·8 |
| HMM 0·1 | 98 ± 5·4 |
| 1 | 99 ± 4·6 |
| 10 | 102 ± 5·0 |
| PMM 0·1 | 108 ± 6·9 |
| 1 | 98 ± 2·8 |
| 10 | 93 ± 2·0 |
| TMM 0·1 | 98 ± 2·4 |
| 1 | 92 ± 3·6 |
| 10 | 99 ± 2·5 |

TABLE II.—*Percentage [$^3$H]TdR uptake by controls after* 120*h exposure to HMM, PMM, TMM, HMPMM and TEM and* 72*h* recovery

| Dose (μg/ml) | [$^3$H]TdR uptake ± s.e. after 120h treatment | [$^3$H]TdR uptake ± s.e. after 72h recovery |
|---|---|---|
| Control | 100 ± 5·3 | 100 ± 4·1 |
| HMM 0·1 | 62 ± 5·7* | 92 ± 8·9 |
| 1 | 70 ± 5·7* | 95 ± 11·6 |
| 10 | 75 ± 3·6* | 84 ± 10·0 |
| 100 | 51 ± 2·3* | 38 ± 2·9* |
| PMM 0·1 | 84 ± 8·2 | 121 ± 11·1 |
| 1 | 68 ± 4·2* | 122 ± 14·3 |
| 10 | 72 ± 4·9* | 115 ± 5·3 |
| 100 | 49 ± 3·3* | 52 ± 5·9* |
| TMM 0·1 | 81 ± 7·4 | 94 ± 15·4 |
| 1 | 122 ± 15·1 | 95 ± 0·4 |
| 10 | 81 ± 5·7 | 90 ± 12·8 |
| 100 | 57 ± 3·8* | 64 ± 6·3* |
| HMPMM 0·1 | 75 ± 3·0* | 70 ± 3·6* |
| 1 | 75 ± 3·9* | 53 ± 4·2* |
| 10 | 42 ± 4·1* | 19 ± 1·5* |
| 100 | 24 ± 4·2* | 13 ± 0·7* |
| TEM 0·1 | 100 ± 8·6 | 14 ± 0·8* |
| 1 | 34 ± 2·9* | 17 ± 0·9* |
| 10 | 30 ± 0·9* | 17 ± 1·6* |
| 100 | 23 ± 2·6* | 19 ± 1·0* |

* $P < 0·01$ Dunnett's *t* test.

FIG. 1.—(A) Percentage of [$^3$H]TdR uptake by controls after 24h exposure to HMM, PMM, TMM, TEM, HMPMM at 100 $\mu$g/ml, formaldehyde at 15 $\mu$g/ml and PMM 100 $\mu$g/ml plus formaldehyde 15 $\mu$g/ml and (B) after 72h recovery.

TEM concentrations of 100 $\mu$g/ml and formaldehyde at about an equimolar concentration for 24 h were active, but HMPMM, TEM and formaldehyde inhibited [$^3$H]TdR uptake significantly more than the other compounds. Formaldehyde at a concentration (15 $\mu$g/ml) equimolar to HMPMM, or formaldehyde plus PMM appear to be more cytotoxic than HMPMM. Shortening the exposure time to 1 h, HMPMM and TEM again caused highly significant reduction of TdR uptake at 100 $\mu$g/ml, respectively 68 and 67% compared to the controls, while HMM did not. When the concentration of the drugs was reduced to 10 $\mu$g/ml the effect was not evident after either 24h or 48h of incubation.

In a third experiment (Table II) the effect of the same methylmelamines and of TEM was studied after 120 h of incubation with the drugs, and after 72 h of recovery in a drug-free medium. [$^3$H]TdR incorporation was greatly reduced at 100 $\mu$g/ml ($P < 0{\cdot}01$) by all the drugs. However, whereas with HMM, PMM and TMM, E cells tended to recover when the drugs were washed out, this effect was not evident with HMPMM and TEM. When the concentrations were reduced to 10, 1 and 0·1 $\mu$g/ml for all the drugs, there was no clear dose-related activity. After 120 h of incubation, TMM lost its activity at 10 $\mu$g/ml and TEM and PMM became inactive at 0·1 $\mu$g/ml; significant reduction of [$^3$H]TdR incorporation by HMM and HMPMM was seen even at 0·1 $\mu$g/ml. When the drugs were washed out for 72 h, no cytotoxic activity was detected for HMM, PMM and TMM previously added at 10 $\mu$g/ml, whereas HMPMM and TEM were still active when previously added at 0·1 $\mu$g/ml. Judging by the effects during the presence of the drug and after its removal, TEM and HMPMM appear to be the most active compounds.

FIG. 2.—Gas chromatograms of medium extracts obtained by nitrogen–phosphorus selective detection. On the left is the chromatogram corresponding to a medium extract to which we added 1 μg/ml of HMM, PMM and TMM; on the right is a chromatogram of the medium containing 1 μg/ml HMM and incubated with E cells for 24 h at 37°C. TEM was used as external standard.

The potential metabolism of HMM by target cells was explored by measuring PMM in the culture medium after incubation of HMM for 24 h at the concentration of 1 μg/ml. No GLC peak for PMM and HMPMM was detectable when the sensitivity was 10 ng/ml (Fig. 2) and HMM did not disappear from the system.

### DISCUSSION

HMM and its metabolites PMM, TMM and HMPMM all inhibit [$^3$H]TdR uptake by E cells, a line derived from a human ovarian tumour. This effect is demonstrable at the very high concentrations of 100 μg/ml for 24 h, or for HMM and HMPMM at the lower concentration of 0·1 μg/ml for 120 h. Concentrations of 10 μg/ml for 24–48 h did not produce any cytotoxicity in our experimental conditions, whereas the 100-fold lower concentration for 120 h did. It therefore appears that the potency of these compounds considerably increases with longer incubation time. This suggests that to improve the clinical efficacy of HMM treatment, it may be more important to keep constant, long-lasting plasma concentrations than to raise the dose.

If we consider the available pharmacokinetic data in humans, we see that after a single oral dose of 120–300 mg/m$^2$ of HMM the plasma peak of HMM is reached in 0·5–4 h and ranges from 0·2 to 20 μg/ml. The plasma level then rapidly declines, followed by a slower phase of disappearance, so that after 12 h the concentration of HMM ranges from 0·02 to 1·2 μg/ml and after 24 h from 0·01 to 0·3 μg/ml (D'Incalci *et al.*, 1978, 1979*b*). On the basis of these pharmacokinetic data we would conclude that the concentrations of HMM we found cytotoxic *in vitro* are comparable with those present in the plasma of patients under treatment with the drug.

Bryan *et al.* (1968) identified all the N-demethylated metabolites in human plasma, and the activity of each was evaluated by Lake *et al.* (1975) who reported that HMM, PMM, TMM had similar potency, but the potency was progressively much lower for the other demethylated compounds. In patients treated with HMM, plasma levels of PMM and TMM reflect those of HMM but are always 2–10 times lower (D'Incalci *et al.*, 1979*b*, and unpublished data).

In our *in vitro* system PMM and TMM were as cytotoxic as HMM, so it is reasonable to assume that the N-demethylated metabolites, or at least PMM and TMM, play a minor role in HMM *in vivo* activity because they are present at lower concentrations. As expected from previous reports, HMPMM is very active on the E-cell line too, but we do not know the relevance of this finding; in fact even though HMPMM was recently found to be a major metabolite of HMM incubated with mice microsomes *in vitro* (Gescher *et al.*, 1979) it has never been identified *in vivo*. Rutty and Connors (1977) reported an increase of formaldehyde concentrations in plasma of HMM-treated mice, which indirectly suggests that HMPMM and other methylols are formed *in vivo*. The study by Kaneko & Lepage (1978) in which line KLN205 from mouse tumours was found sensitive to HMM *in vivo* but not *in vitro*, also suggests that some metabolites other than HMM could be responsible for the anticancer effect. The release of formaldehyde has been offered to explain the mode of action of HMM, but reportedly the formaldehyde inhibitor semicarbazide did not prevent HMPMM toxicity (Rutty & Connors, 1977) though a more recent report claims that it did reverse toxicity at a very low HMPMM concentration (Rutty & Harrap, 1978). Our data also suggest that formaldehyde could be responsible for HMPMM activity; the ability of semicarbazide to reverse HMPMM cytotoxicity also in our system is currently being investigated.

We failed to find any metabolism of HMM by E cells, which might explain the cytotoxicity observed in terms of activation to HMPMM. In spite of our negative results, however, this hypothesis cannot be excluded, as the sensitivity of our method of detecting PMM or HMPMM (10 ng/ml) may not be sufficient considering the relatively small number of cells in our system.

Further study is warranted to confirm these data on primary cultures of ovarian cancer, where in some cases it is possible to predict the clinical efficacy (Salmon *et al.*, 1978) and to establish whether HMM and its metabolites are cytotoxic at even lower concentrations if the contact time is further prolonged.

### REFERENCES

BOLIS, G., D'INCALCI, M., BELLONI, C. & MANGIONI, C. (1979) Hexamethylmelamine in ovarian cancer resistant to cyclophosphamide and adriamycin. *Cancer Treat. Rep.*, **63**, 1375.

BONOMI, P. D., MLADINEO, J., MORRIN, B., WILBANKS, G., JR & SLAYTON, R. E. (1979) Phase II trial of hexamethylmelamine in ovarian carcinoma resistant to alkylating agents. *Cancer Treatment Rep.*, **63**, 137.

BLUM, R. H., LIVINGSTON, R. B. & CARTER, S. K. (1973) Hexamethylmelamine. A new drug with activity in solid tumors. *Eur. J. Cancer*, **9**, 195.

BRYAN, G. T., WORZALLA, J. F., GORSKE, A. L. & RAMIREZ, G. (1968) Plasma levels and urinary excretion of hexamethylmelamine following oral administration to human subjects with cancer *Clin. Pharmacol. Ther.*, **9**, 777.

DEMILO, A. B. & BOŘKOVEC, A. B. (1968) Insect chemosterilants. VII Oxidative degradation of hexamethylmelamine. *J. Med. Chem.*, **11**, 961.

D'INCALCI, M., BOLIS, G., MANGIONI, C., MORASCA, L. & GARATTINI, S. (1978) Variable oral absorption of hexamethylmelamine in man. *Cancer Treat. Rep.*, **62**, 2117.

D'INCALCI, M., MORAZZONI, P. & PANTAROTTO, C. (1979*a*) Gas chromatographic determination of hexamethylmelamine in mouse plasma. *Anal. Biochem.*, **33**, 441.

D'INCALCI, M., SESSA, C., BELLONI, C., MORASCA, L. & GARATTINI, S. (1979*b*) Hexamethylmelamine (HMM) and pentamethylmelamine (PMM) levels in plasma and ascites after oral administration to ovarian cancer patients. *Proc. Am. Assoc. Cancer Res.*, **20**, 46.

GESCHER, A., D'INCALCI, M., FANELLI, R. & FARINA, P. (1980) N-hydroxymethylpentamethylmelamine, a major *in vitro* metabolite of hexamethylmelamine. *Life Sci.*, **26**, 147.

HEERE, L. J. & DONNELLY, S. T. (1971) Antitumor activity of hexamethylmelamine and 4(5)-(3,3-dimethyl-1-triazeno)-imidazole-5(4)-carboxamide. *Proc. Am. Assoc. Cancer Res.*, **12**, 101.

JOHNSON, B. L., FISHER, R. I., BENDER, R. A., DEVITA, V. T., JR, CHABNER, B. A. & YOUNG, R. C. (1978) Hexamethylmelamine in alkylating agent-resistant ovarian carcinoma. *Cancer*, **42**, 2157.

KANEKO, T. & LEPAGE, G. A. (1978) Growth characteristics and drug responses of a murine lung carcinoma *in vitro* and *in vivo*. *Cancer Res.*, **38**, 2084.

LAKE, L. M., GRUNDEN, E. E. & JOHNSON, B. M. (1975) Toxicity and antitumor activity of hexamethylmelamine and its N-demethylated metabolites in mice with transplantable tumors. *Cancer Res.*, **35**, 2858.

LEGHA, S. S., SLAVIK, M. & CARTER, S. K. (1976) Hexamethylmelamine. An evaluation of its role in the therapy of cancer. *Cancer*, **38**, 27.

LEGHA, S. S., SLAVIK, M., LIVINGSTON, R. B. & CARTER, S. K. (1974) Hexamethylmelamine (NSC 13875). *Clin. Brochure, Natl Cancer Inst.*

RUTTY, C. J. & CONNORS, T. A. (1977) *In vitro* studies with hexamethylmelamine. *Biochem. Pharmacol.*, **26**, 2385.

RUTTY, C. J., CONNORS, T. A., NGUYEN-HOANG-NAM, DO-CAO-THANG & HOELLINGER, H. (1978) *In vivo* studies with hexamethylmelamine. *Eur. J. Cancer*, **14**, 713.

RUTTY, C. J. & HARRAP, K. R. (1978) Methylmelamines: Demethylation and antitumour activity. *Br. J. Cancer*, **39**, 187.

SALMON, S. E., HAMBURGER, A. W., SOEHNLEN, B., DURIE, B. G. M., ALBERTS, D. S. & MOON, T. E. (1978) Quantitation of differential sensitivity of human-tumor stem cells to anticancer drugs. *N. Engl. J. Med.*, **298**, 1321.

TAKITA, H. & DIDOLKAR, M. S. (1974) Effect of hexamethylmelamine (NSC-13875) on small cell carcinoma of the lung (Phase II Study). *Cancer Chemother. Rep.*, **58**, 371.

WILSON, W. L., SCHROEDER, J. M., BISEL, H. F., MRAZEK, R. & HUMMEL, R. P. (1969) Phase II study of hexamethylmelamine (NSC 13875). *Cancer*, **23**, 132.

WORZALLA, J. F., JOHNSON, B. M., RAMIREZ, G. & BRYAN, G. T. (1973) N-Demethylation of the antineoplastic agent hexamethylmelamine by rats and man. *Cancer Res.*, **33**, 2810.

WORZALLA, J. F., KAIMAN, B. D., JOHNSON, B. M., RAMIREZ, G. & BRYAN, G. T. (1974) Metabolism of hexamethylmelamine-ring-$^{14}C$ in rats and man. *Cancer Res.*, **34**, 2669.